# Histopathological patterns and topographic distribution of endometriosis: A 10-year retrospective study at a South African tertiary institution

**DOI:** 10.4102/jcmsa.v4i1.421

**Published:** 2026-10-19

**Authors:** Florence L. Masha, Nosipho M. Thobakgale, Moshawa C. Khaba

**Affiliations:** 1 Department of Anatomical Pathology, School of Medicine, Sefako Makgatho Health Sciences University, Pretoria, South Africa; 2 Department of Anatomical Pathology, Dr George Mukhari Academic Laboratory, National Health Laboratory Service, Pretoria, South Africa

**Keywords:** endometriosis, histopathological diagnostic frequency, histopathology, topographic distribution, South Africa

## Abstract

**Background:**

Endometriosis affects approximately 190 million women globally; however, despite this, epidemiological data on endometriosis from Africa remain scarce. Understanding regional disease patterns, histopathological characteristics, and anatomical distribution is essential for improving diagnostic accuracy and clinical management.

**Methods:**

A retrospective cross-sectional study was conducted using archival data from the Department of Anatomical Pathology at the Dr George Mukhari Academic Laboratory, Ga-Rankuwa, South Africa, from 01 January 2012 to 31 December 2021. Histopathologically confirmed cases of endometriosis were identified using SNOMED codes. Demographic and clinical data were analysed using Stata 14, with Chi-square and Fisher’s exact tests to assess associations (*p* < 0.05).

**Results:**

Of 93 804 female specimens examined, 156 cases of endometriosis were confirmed (histopathological diagnostic frequency = 0.166%). The mean age at diagnosis was 36.97 years. The most common histopathological pattern (60.3%) demonstrated all three classic features. The ovary was the most frequently affected site (36.5%). Synchronous involvement at multiple sites occurred in 9.6% of cases, predominantly affecting the adnexa. Endometriotic cysts were identified in 28.8% of patients. Cervical endometriosis was associated with older age (≥ 50 years), although based on a small number of cases (*p* = 0.008). Associated lesions were identified in 24.4% of the cases.

**Conclusion:**

These findings contribute to the limited epidemiological data on endometriosis in Africa and emphasise the need for heightened clinical awareness, particularly for extrauterine manifestations that may present diagnostic challenges.

**Contribution:**

This study contributes to the limited South African data by providing detailed insights into the histopathological diagnostic frequency, anatomical distribution and histopathological patterns, enhancing understanding of disease patterns.

## Introduction

Endometriosis is a major global public health issue, with an estimated 190 million women of reproductive age affected worldwide. The World Endometriosis Research Foundation (WERF) estimates the prevalence to be 1 in 10 women globally.^1^ However, epidemiological data from Africa are limited, and very few representative studies have been conducted to establish the true burden of disease on this continent. A study conducted in South Africa by Mashele et al. (2022) found only 34 cases over a 2-year period,^2^ underscoring the urgent need for nationally representative studies to clarify regional epidemiological patterns and expand global understanding of this condition.

The pathogenesis of endometriosis is complex and multifactorial, with genetic, epigenetic and environmental components. Several aetiological theories have been postulated concerning retrograde menstruation, benign metastasis, coelomic metaplasia and stem cell theory as possible explanations for disease development; however, none can fully explain all clinical presentations.^3,4^ Reproductive factors such as parity, age at menarche, characteristics of the menstrual cycle and use of oral contraceptives have consistently been associated with an increased risk of endometriosis, suggesting that hormonal influences may play a central role in susceptibility to this disease as well as its progression.^3,4^

Diagnosis of endometriosis requires an accurate, multimodal approach that integrates clinical evaluation, imaging techniques, laparoscopic visualisation and histopathological confirmation. The clinical and radiological features of endometriosis are highly heterogeneous; hence, timely, accurate diagnosis may be complicated. Histopathologically, the diagnosis of endometriosis requires the presence of at least two of the following three features: Endometrial epithelium and/or glands, endometrial stroma and chronic haemorrhage (repeated cyclical bleeding within ectopic endometrial tissue), evidenced by haemosiderin pigment deposition.^2^ Though histopathological examination remains the gold standard for definitive diagnosis, it is not without challenges, even under microscopic evaluation, because of differences in glandular and stromal components, including endometrial stromal decidualisation, haemorrhage and fibrosis, among other changes described in the literature.^2,4^

Current guidelines for treatment in South Africa recommend a stepwise approach to management of endometriosis where medical therapy is instituted first-line and surgical treatment reserved for those who are refractory or have complications like severe pain, infertility or organ dysfunction.^3,5^ Medical therapy follows a stepwise approach, consisting of first- and second-line therapy. The first-line therapy consists of combined oral contraceptives (COCs) and progestins. In case of first-line failure, the second line consists of gonadotropin-releasing hormone agonists. Non-steroidal anti-inflammatory drugs are adjunctive for symptomatic relief of pain. In patients with pain from deep infiltrating endometriosis resistant to surgical or medical therapy, aromatase inhibitors can be used as an additional option. This conservative line reflects both resource considerations and an attempt at preserving fertility among affected women within reproductive age.^6^

Given the clinical importance of endometriosis and its effects on quality of life, fertility and healthcare systems, comprehensive information on its histopathological diagnostic frequency, histopathological features and anatomical distribution in South African populations remains undocumented. Understanding local patterns of this disease, along with related pathological findings and site-specific manifestations, is needed to improve diagnostic accuracy, ensure reliable treatment protocols and identify areas requiring greater clinical vigilance.

Therefore, this study sought to establish the frequency of histologically confirmed endometriosis at our tertiary institution and to characterise the anatomical sites and histopathological patterns.

## Research methods and design

This retrospective, cross-sectional, descriptive study included all cases with a histopathological diagnosis of endometriosis at the Department of Anatomical Pathology, Dr George Mukhari Academic Laboratory (DGMAL), Ga-Rankuwa, South Africa, from 01 January 2012 to 31 December 2021.

### Case identification and selection

We identified endometriosis cases from the National Health Laboratory Service (NHLS) laboratory information system using a combined strategy. Firstly, we performed structured retrieval using relevant Systematised Nomenclature of Medicine (SNOMED) morphology (M) and topography (T) codes, selected a priori based on the diagnostic entities of interest. We used these codes to extract histopathology records within the defined study period. Secondly, to minimise missed cases as a result of coding variability or incomplete coding, we conducted a supplementary free-text search of pathology reports using predefined keywords and synonyms corresponding to the study condition, such as ‘endometriosis’, ‘endometrioma’ and ‘endometriotic cyst’. We merged the results from both approaches and removed duplicate records.

All diagnoses included in the dataset were based on original histopathology reports issued by qualified NHLS pathologists in routine clinical practice. Within standard NHLS diagnostic workflows, cases may undergo intradepartmental review, including peer discussion or second review by another pathologist in diagnostically challenging or complex cases, as part of routine quality assurance processes. For this study, we systematically performed additional independent re-review of all cases; when clarification was needed during data extraction or analysis, selected cases were re-examined by a study pathologist to confirm diagnostic categorisation.

When multiple specimens were obtained from the same patient during a single clinical episode, we linked them using available patient identifiers and accession numbers. For frequency calculations, we counted each patient once. However, when endometriosis involved multiple anatomical sites, we documented and included all affected sites in the topographic analysis.

Cases were excluded if the diagnosis of endometriosis could not be confirmed following histopathological review, or if tissue material was unavailable for reassessment, or if an archived formalin-fixed paraffin-embedded (FFPE) tissue block could not be retrieved in the absence of tissue slides.

### Data collection

We extracted demographic and clinicopathological data from pathology request forms, laboratory records and archived reports. Variables collected included patient age at diagnosis, specimen type, anatomical site of involvement, final diagnosis, present diagnostic histopathological features and associated or concomitant pathological lesions.

The anatomical location of endometriosis was classified according to the primary site of involvement documented in the pathology report. Cases with insufficient information regarding the site of origin were categorised as ‘unknown’.

### Histopathological review

Archived haematoxylin and eosin (H&E)-stained slides from all eligible cases were retrieved and independently reviewed by the principal investigator and two specialist anatomical pathologists. Histological assessment focused on endometrial glands, endometrial stroma, chronic haemorrhage, haemosiderin-laden macrophages, chronic inflammation and any associated pathological findings.

When original slides were unavailable, damaged or unsuitable for review, FFPE tissue blocks were retrieved from the archives and recut at 4 µm for repeat H&E staining. In total, 15 cases were recut because H&E slides were unavailable for review or were faded or broken.

The diagnosis of endometriosis was confirmed according to established histopathological criteria, requiring the presence of at least two of the following three features: Endometrial-type glands, endometrial-type stroma and evidence of chronic haemorrhage (repeated cyclical bleeding within ectopic endometrial tissue), including haemosiderin deposition and/or haemosiderin-laden macrophages.^2^ We then categorised histopathological patterns according to the combination of these diagnostic features.

### Data analysis

Data were captured in Microsoft Excel and analysed using Stata version 14 (StataCorp LLC, College Station, TX, United States).

Continuous variables were summarised using means and ranges, while categorical variables were expressed as frequencies and percentages.

Associations between anatomical site and age category, as well as between endometriotic cyst formation and anatomical site, were assessed using Fisher’s exact test because of the small number of observations in certain categories. We defined statistical significance as a two-sided *p*-value < 0.05.

### Ethical considerations

Ethical clearance to conduct this study was obtained from the Sefako Makgatho University Research Ethics Committee (No: SMUREC/ M/186/2023:PG).

## Results

### Frequency of endometriosis

Of 93 804 cases among specimens from female patients during the study period, 156 had histopathologically confirmed endometriosis. Figure 1 depicts the diagnostic trend over a 10-year period.

**FIGURE 1:**
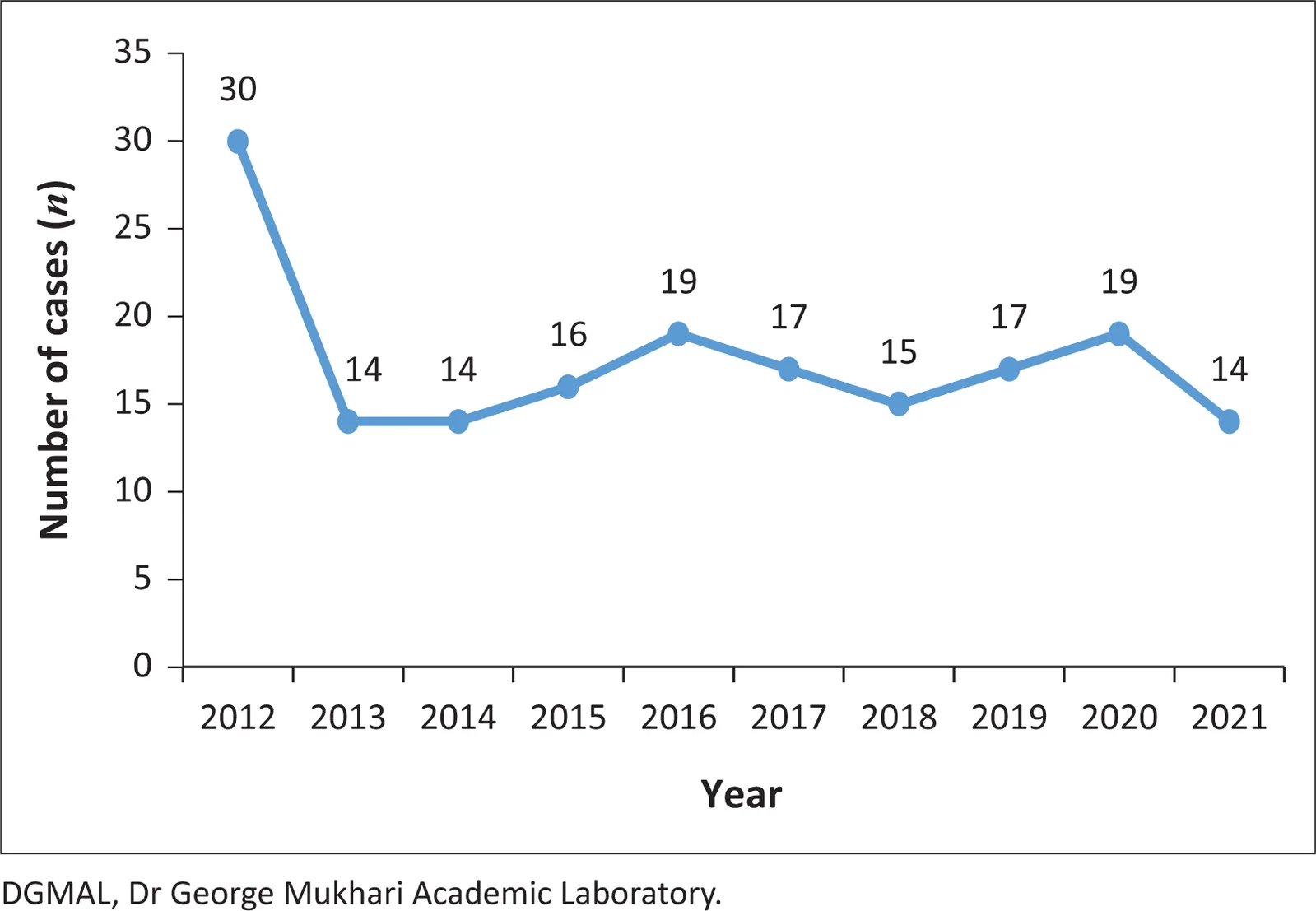
The trend of endometriosis diagnosis at Dr George Mukhari Academic Laboratory over 10-years.

### Age distribution of the cases

We identified 156 cases of histologically confirmed endometriosis during the study period. Age data were available for 155 patients (Figure 2). The mean age of patients with endometriosis was 36.97 ± 8.60 years (range 19–70 years). The largest proportion of cases occurred in women aged 30–34 years (23.9%), followed by those aged 35–39 years (20.6%) and 40–44 years (19.4%). Only four cases (2.6%) occurred in women younger than 25 years, while nine cases (5.8%) were identified in women aged 50 years and older (Figure 2).

**FIGURE 2:**
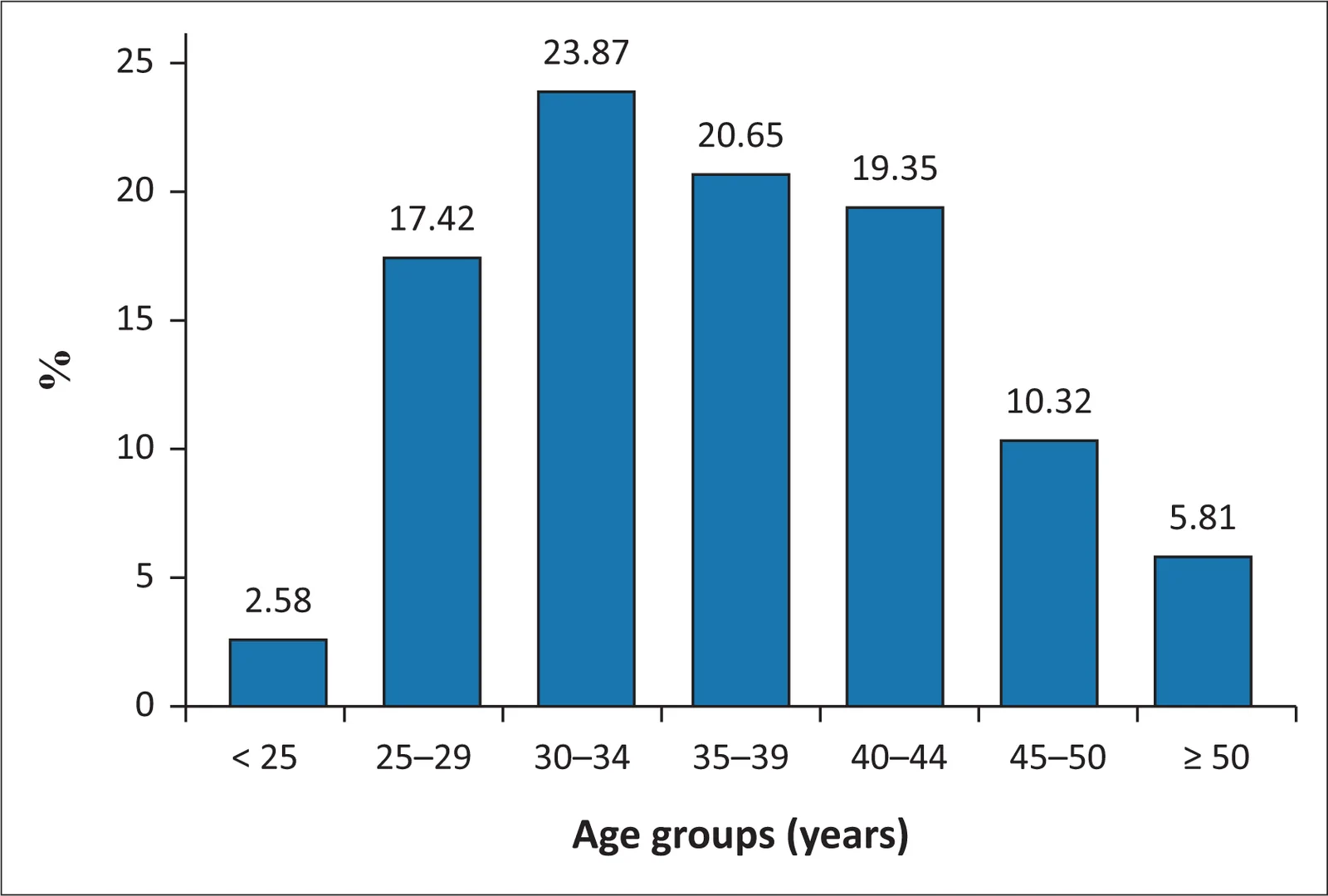
Age distribution of the cases with endometriosis.

### Histopathological patterns

Four histopathological patterns were identified. The most common pattern comprised endometrial glands, endometrial stroma and chronic haemorrhage, accounting for 94 cases (60%) (Figure 3 and Figure 4). Endometrial glands with endometrial stroma were observed in 29 cases (19%) (Figure 3 and Figure 4), followed by endometrial glands with chronic haemorrhage in 20 cases (13%) (Figure 3 and Figure 4). The least frequent pattern consisted of endometrial stroma with chronic haemorrhage, identified in 13 cases (8%) (Figure 3 and Figure 4).

**FIGURE 3:**
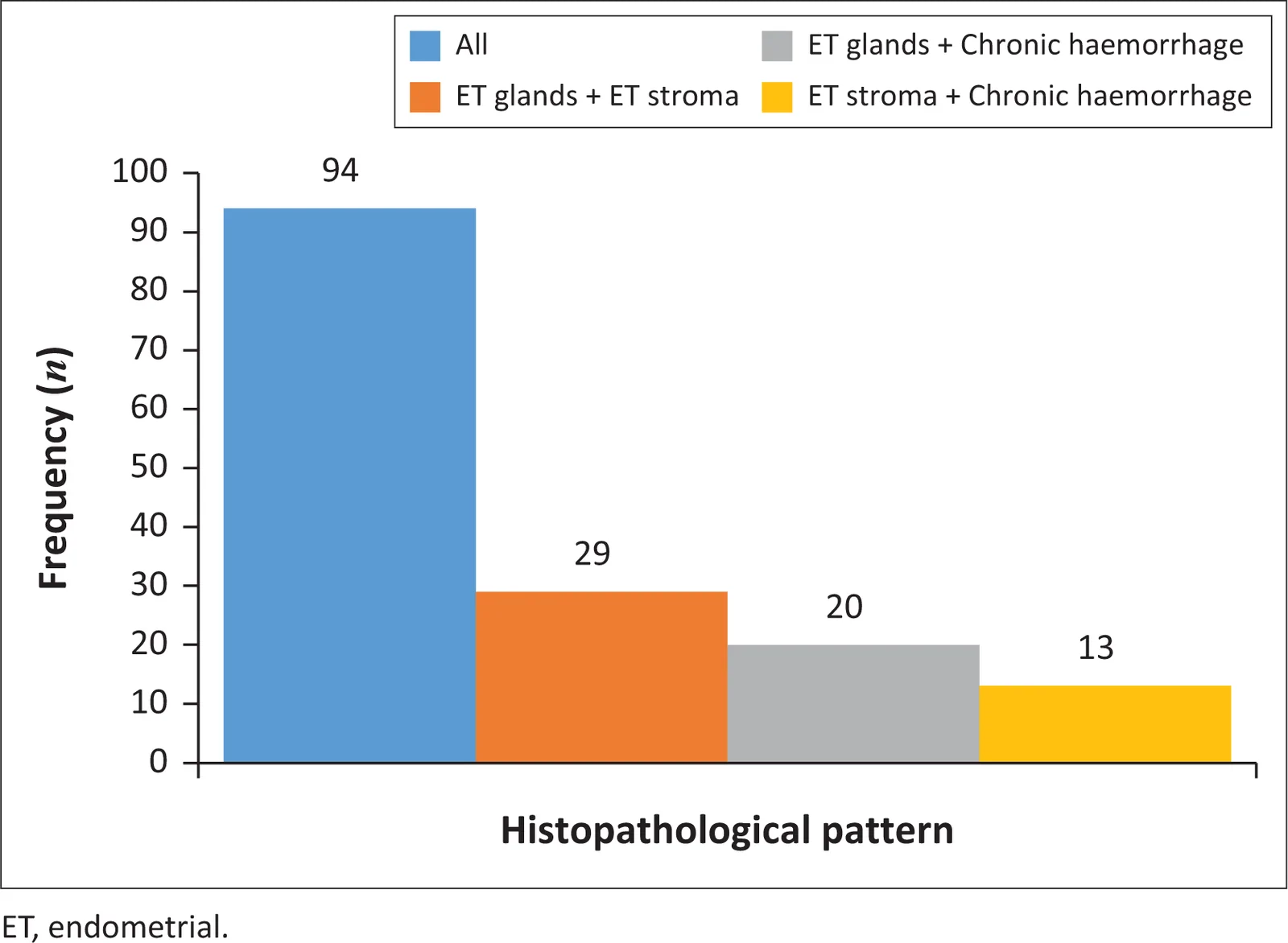
Histopathological patterns of endometriosis.

**FIGURE 4:**
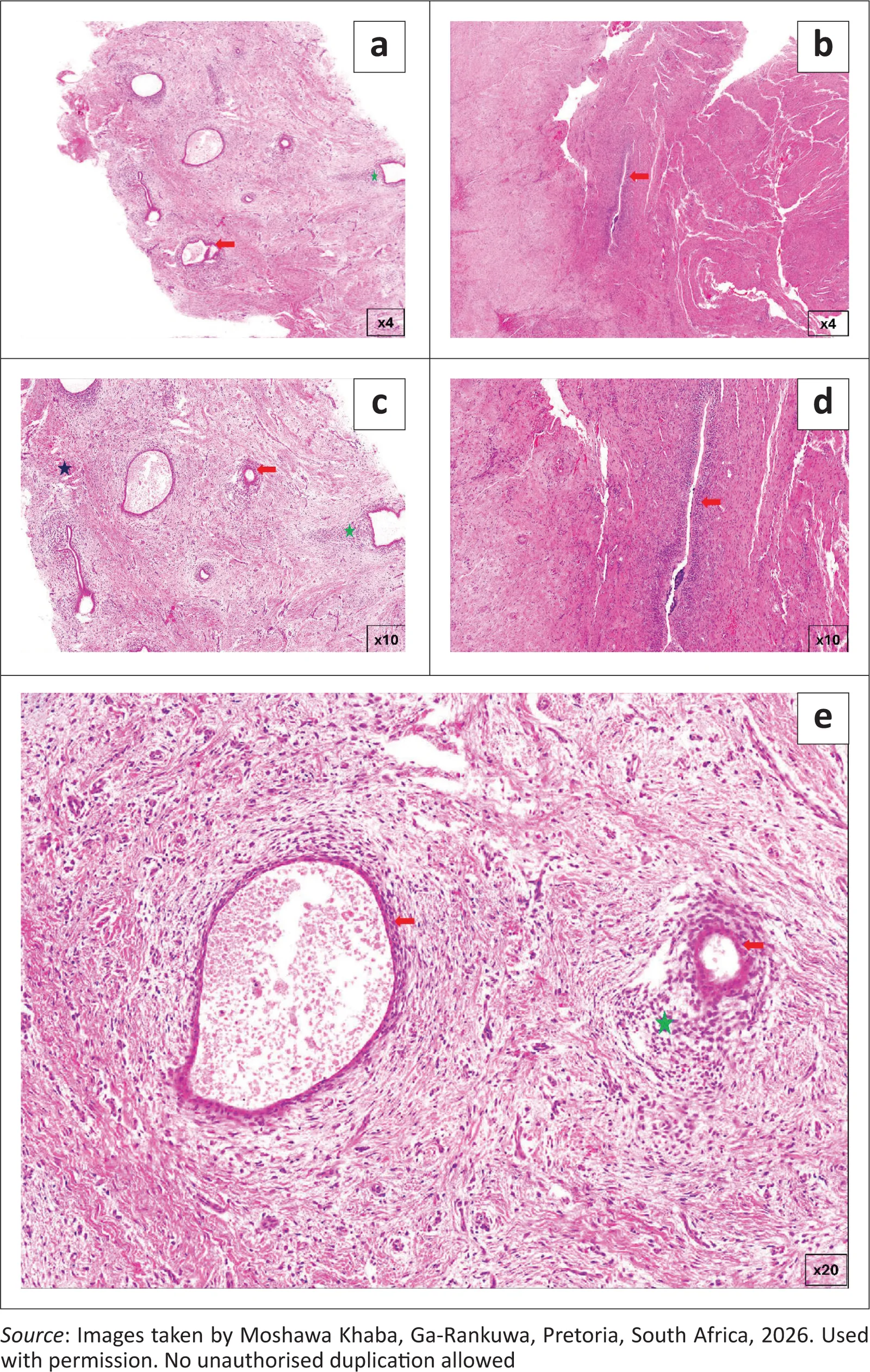
(a–e) show endometrial glands, stroma and haemorrhage at different magnifications. Red arrows show endometrial-type glands; Green stars show endometrial-type stroma; black star shows haemorrhage.

### Topographic sites of endometriosis

The ovary was the most frequently affected site, accounting for 57 cases (36.5%), followed by the umbilicus (35 cases; 22.4%), abdominal wall (12 cases; 7.7%) and fallopian tube (12 cases; 7.7%). Cervical involvement was identified in eight cases (5.1%). Less commonly affected sites included Caesarean section scars, skin, large bowel, appendix, omentum and other extra-pelvic locations. The anatomical site could not be determined in 11 cases (7.1%) (Table 1).

**TABLE 1:** Topographic sites of endometriosis (*N* = 156).

Topographic site	Number of cases (*n*)	%
Ovary	57	36.5
Umbilicus	35	22.4
Abdominal wall	12	7.7
Fallopian tube	12	7.7
Cervix	8	5.1
Caesarean section scar	4	2.6
Skin NOS	4	2.6
Scar NOS	2	1.3
Large bowel	2	1.3
Appendix	2	1.3
Omentum	2	1.3
Other†	5	3.2
Unknown	11	7.1
Total	156	100

NOS, not otherwise specified.

† , Other: Peritoneum; Pouch of Douglas; Round ligament; Vagina.

Endometriosis involving more than one anatomical site was identified in 15 patients (9.6%). The most common pattern was bilateral ovarian involvement, observed in seven cases (46.7%). Concurrent involvement of the fallopian tube and ovary or bilateral ovaries was identified in four cases (26.7%), while cervical and ovarian or bilateral ovarian involvement occurred in two cases (13.3%). Less frequent combinations included cervical and fallopian tube involvement (one case; 6.7%) and fallopian tube and round ligament involvement (one case; 6.7%) (Figure 5).

**FIGURE 5:**
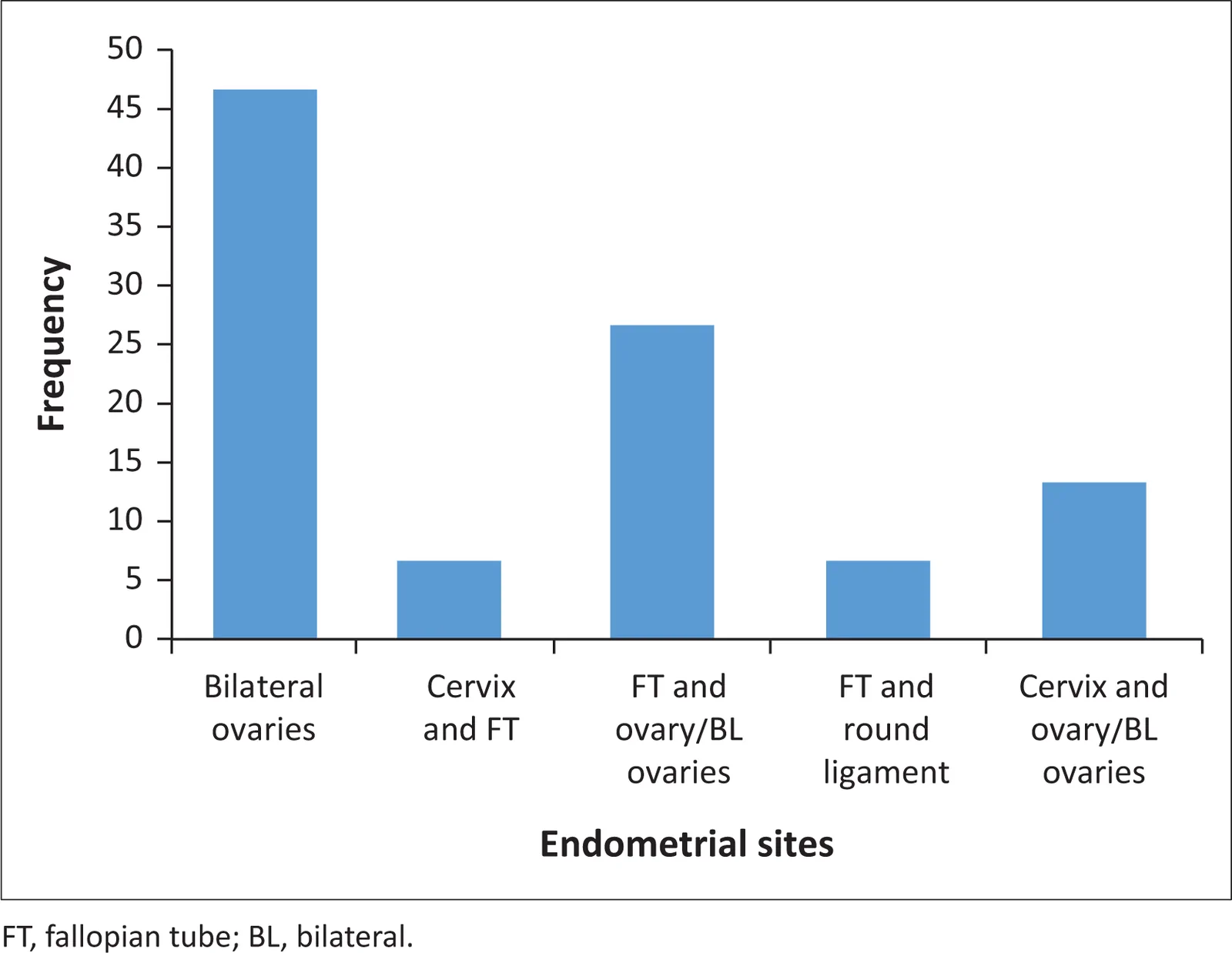
Distribution of multi-site endometriosis (*N* = 15).

### Topographic sites of endometriosis by age

Table 2 details the relationship between the topographical data and age. Fischer’s exact test revealed a statistically significant connection (*p* = 0.008) between cervical topography and patient age, highlighting the cervix as the predominant site of endometriosis in older women aged 50 and above. Conversely, other sites showed no significant association with age. The most common sites of endometriosis in patients aged ≤ 49 years are the ovaries and umbilicus, whereas in patients aged ≥ 50 years, the ovaries, cervix and fallopian tubes are often affected.

**TABLE 2:** Association between age and the top six topographic sites of endometriosis (*N* = 127).

Topographic site	Total		Age (years)													
			< 25		25–29		30–34		35–39		40–44		45–49		≥ 50	
	*n*	%	*n*	%	*n*	%	*n*	%	*n*	%	*n*	%	*n*	%	*n*	%
Ovary	53	41.7	1	1.9	11	20.8	14	26.4	14	26.4	7	13.2	4	7.5	2	3.8
Umbilical region	39	30.7	1	2.6	5	12.8	9	23.1	9	23.1	8	20.5	6	15.4	0	0
Abdominal	17	13.4	1	5.9	3	17.6	5	29.4	3	17.6	3	17.6	1	5.9	1	5.9
Fallopian tubes	6	4.7	0	0	0	0	0	0	0	0	4	66.7	0	0	2	33.3
Bilateral ovaries	7	5.5	1	14.3	3	42.9	2	28.6	0	0	0	0	0	0	1	14.3
Cervix	5	3.9	-	0	0	0	0	0	0	0	2	40	1	20	2	40

Note: This subset analysis is restricted to the six most frequently affected topographic sites and to cases with complete age data; site counts therefore differ from the full cohort reported in Table 1, which includes all 156 cases and all anatomical sites.

Table 2 shows that ovarian endometriosis was the most common site (41.7%) (Figure 6), especially in women aged 30–39, highlighting its oestrogen-dependent nature. Umbilical endometriosis followed at 30.7%, with no cases in women aged ≥ 50, suggesting a decline with age. Fallopian tube endometriosis was noted primarily in women aged 40–44 (66.7%), while cervical endometriosis affected 40% of those ≥ 50, supporting the statistically significant age association identified on Fisher’s exact test (*p* = 0.008). Bilateral ovarian involvement was most prevalent in younger women aged 25–34 (71.5%) (Figure 6), indicating a possible aggressive disease onset early in reproductive years.

**FIGURE 6:**
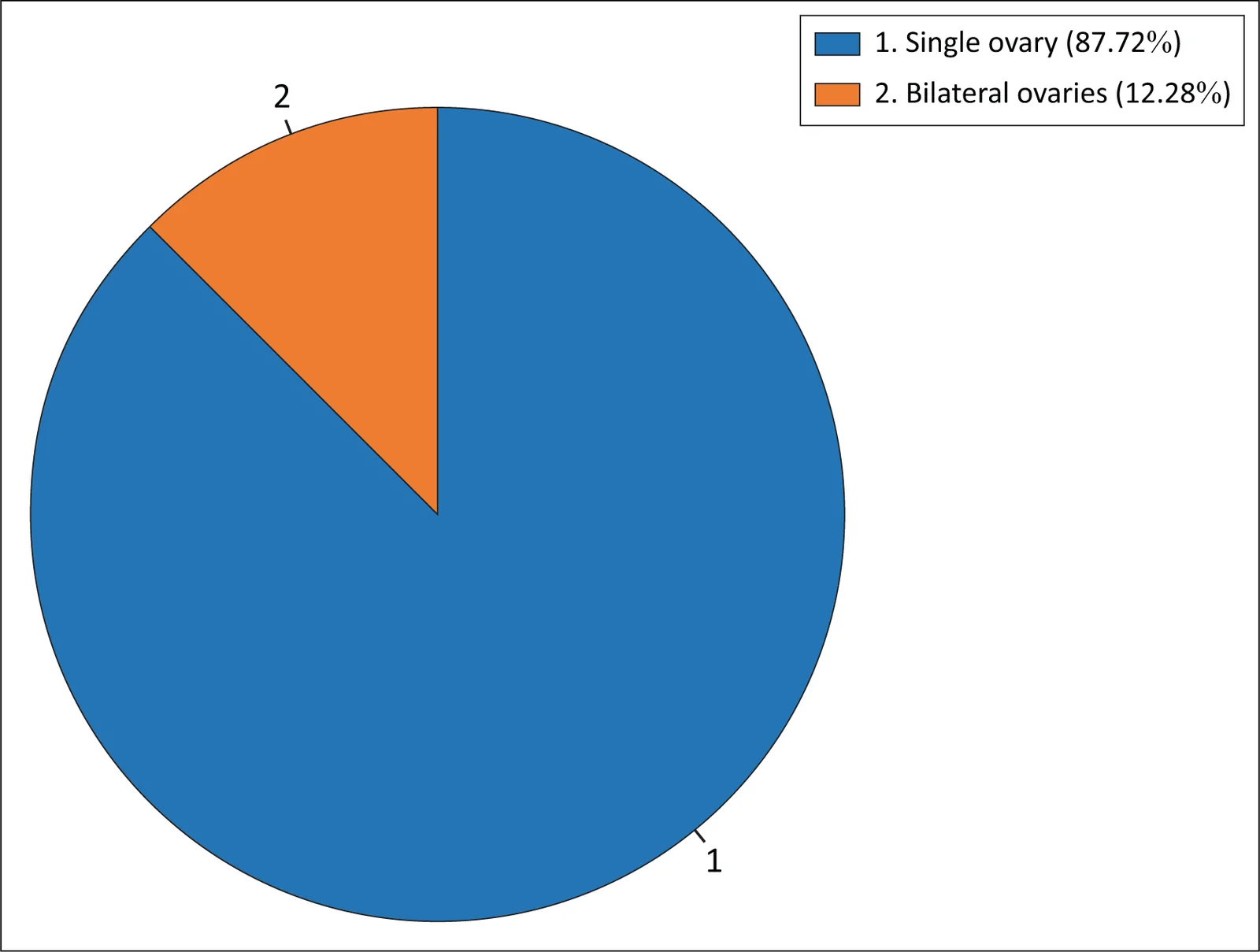
Ovarian involvement by endometriosis.

### Endometriotic cysts

We observed endometriotic cysts in 45 (28.8%) patients. We assessed the association between these data and topographical data using Fisher’s exact test and found it significant (*p*-value = 0.001). This indicates that the ovary was the most common site for endometriotic cyst development. Seven cases had bilateral ovarian endometriotic cysts.

### Lesions associated with endometriosis

Benign gynaecological pathology predominated in the 38 cases with associated lesions. Uterine leiomyoma was the most frequent finding (47.4%), followed by adenomyosis (15.8%) and eutopic endometrial hyperplasia (7.9%). The remaining benign lesions were serous cystadenoma, mature cystic teratoma and mucinous cystadenoma. We found pre-malignant disease in two cases. Malignant tumours identified comprised squamous cell carcinoma and low-grade endometrioid adenocarcinoma, each representing 5.3% of associated findings. The coexistence of endometriosis with benign and malignant pathology in this cohort underscores the importance of a detailed histopathological assessment of all resected specimens (Table 3).

**TABLE 3:** Lesions associated with endometriosis (*N* = 38).

Associated lesions	Number of cases (*n*)	%
**Benign lesions**		
Uterine leiomyoma	18	47.4
Adenomyosis	6	15.8
Eutopic endometrial hyperplasia	3	7.9
Serous cystadenoma	2	5.3
Mature cystic teratoma	2	5.3
Mucinous cystadenoma	1	2.6
**Pre-malignant lesion**		
High-grade squamous intraepithelial neoplasia	2	5.3
**Malignant lesions**		
Squamous cell carcinoma	2	5.3
Low-grade endometrioid adenocarcinoma (FIGO I)	2	5.3

FIGO, International Federation of Gynaecology and Obstetrics.

## Discussion

### Prevalence of endometriosis: The African case in perspective

This study reported a histopathological diagnostic frequency of 156 cases among 93 804 specimens from female patients received at our institution over the 10-year study period. Endometriosis frequency estimates from the African continent are heterogeneous, ranging from 0.2% to 48%, depending on study design, population selection and diagnostic methodology.^7,8^

It has been historically assumed that endometriosis is rare among women of African descent; thus, this belief keeps many women from receiving timely diagnoses and accessing specialised care. It has also led to very little research being conducted on African populations.^7^ Current evidence refutes this paradigm by showing that the real prevalence of endometriosis in Africa is probably much higher than what has been documented, with average diagnostic delays reported at between 7 years and 10 years.^9^ Therefore, our institution’s low histopathological diagnostic frequency may reflect underdiagnosis rather than genuine rarity, compounded by limited access to advanced imaging and laparoscopic capabilities.

Studies using laparoscopic visualisation alone reported prevalence rates as high as 48.1%. However, these estimates should be interpreted with caution, as laparoscopy without histopathological confirmation has very high false-positive and false-negative rates.^8,10^ Nigerian studies with histopathological confirmation have reported lower prevalence rates, comparable to those observed in the present study, further supporting histopathological examination as the gold standard for diagnosing endometriosis.^8,11^ The global prevalence of histologically confirmed endometriosis in reproductive-age women is about 10%,^12^ and it is substantially more than what we have observed.

A recent narrative review revealed substantial differences: Health insurance databases report endometriosis affecting only ~1% of women, while clinical studies find it at 6.8%, population-based surveys put it at 6.6%, and symptomatic patient cohorts report a staggering 21%.^11^ These results clearly highlight the pivotal roles of denominator choice and diagnostic strictness in epidemiological studies. Our study yields a more conservative, yet methodologically sound estimate of histopathological diagnostic frequency that avoids the selection bias inherent in fertility clinic-based or symptomatic cohort studies.

### Age distribution and life course patterns

The mean age at diagnosis was 36.97 years (range: 19–70 years), consistent with the literature describing a peak in endometriosis diagnoses in the third and fourth decades of life.^2,8^ The highest proportion of cases was in the 30–34-year age group (23.9%) and was sustained through the fourth decade, reflecting the natural history of endometriosis as an oestrogen-dependent condition affecting women during their reproductive years.^12^

Notably, 5.81% of diagnoses were made in women aged 50 years and older; this fact challenges the traditional paradigm of endometriosis as a disease that only affects younger women who are of reproductive age. Though less common, postmenopausal endometriosis has been increasingly recognised and this may be because of residual ovarian function or exogenous oestrogen exposure through hormone replacement therapy or aromatase activity within the endometriotic lesions themselves.^7,9^

Endometriosis in postmenopausal women has particular clinical significance since there is an increased risk for malignant transformation in this group; rates are estimated at 1% – 2.5% against approximately 1% for premenopausal women.^13,14^ The decline may reflect changes in referral patterns, healthcare infrastructure, gynaecological practice patterns or surgical case mix rather than a real decrease in disease prevalence. Perhaps increased awareness and earlier intervention have reduced the proportion of cases that become so severe as to require surgical management and histopathological evaluation.

### Histopathological patterns: Diagnostic implications and challenges

The predominant histopathological pattern in our study, the classic triad of endometrial glands, endometrial stroma and chronic haemorrhage (60.3%), is consistent with established diagnostic criteria for endometriosis and reflects complete preservation of the architecture of normal residual functional endometrium at ectopic sites.^2^ Current pathological diagnostic criteria require at least two out of three microscopic features: Glands resembling those found normally within the uterus, endometrial-type stroma resembling that found normally within the uterus or chronic haemorrhage evidenced by hemosiderin-laden macrophages.

The varying frequencies of incomplete patterns, endometrial glands with endometrial stroma (18.6%), endometrial glands with chronic haemorrhage (12.8%), and endometrial stroma with chronic haemorrhage (8.3%), emphasise the critical challenges in diagnosis. Ectopic foci are characterised by glandular epithelial cells surrounded by periglandular stroma filled with round or oval cells with little cytoplasm and inflammatory elements that maintain a pro-inflammatory microenvironment, alongside neoangiogenesis that supports lesion growth and survival.^9,12^ Long-standing lesions develop progressive fibrosis and stromal obliteration; hence, recognition of classical features becomes more difficult.

Stromal endometriosis, the presence of endometrial stroma without its glands, poses a specific diagnostic challenge and may be mistaken for nonspecific fibrous tissue or an inflammatory infiltrate. In such difficult cases, CD10 immunohistochemistry has become an extremely useful diagnostic tool because this marker is expressed sensitively and specifically by endometrial stromal cells at both eutopic and ectopic sites.^15^ When endometrial-type glands and stroma are few or absent, staining with CD10 can confirm the diagnosis by proving that the stromal components have an origin from the endometrium.

The metaplastic phenomenon of glandular inclusions, such as tubal, mucinous, squamous and hobnail changes, adds another layer of morphological unrecognition and may confuse its diagnosis with primary neoplastic processes. Atypical endometriosis is also characterised by an enlarged epithelium with abundant eosinophilic cytoplasm, stratification and nuclear hyperchromasia, representing an important intermediate stage between benign endometriosis and endometriosis-associated ovarian carcinomas (EAOC), especially the clear cell and endometrioid subtypes.^14,16,17^

### Topographic distribution: Clinical correlates

The ovary was the most common site involved in our series (36.5%), consistent with global reports, in which ovarian endometriosis is the most common form of the disease.^2^ The predominance of ovarian involvement may be explained by the ovary’s proximity to the fallopian tubes, which facilitates retrograde menstrual seeding, as well as by cortical invaginations and a hormonally favourable microenvironment that support the implantation, survival and proliferation of ectopic endometrial tissue.^18^

A large number of extragonadal sites were found, especially the umbilicus (22.4%), abdominal wall (7.7%) and a caesarean section scars (2.6%). This highlights that endometriosis can occur in many different parts of the body. Umbilical endometriosis is not common, but it often happens after some prior surgery on the stomach or because of spontaneous implantation. Scar endometriosis following Caesarean section or other pelvic surgical procedures is believed to result from the inadvertent transplantation of endometrial tissue into the surgical wound, where it subsequently implants, survives and proliferates under hormonal stimulation.

The finding that it commonly affects multiple anatomical sites (9.6% of cases), primarily involving the adnexa with bilateral ovarian disease in seven patients, indicates that endometriosis has a systemic nature and backs up theories about haematogenous or lymphatic dissemination rather than just simple retrograde menstruation. The involvement of the fallopian tube (7.7%) and cervical endometriosis (5.1%) are less common but clinically significant forms that might be underrecognized or misdiagnosed as other diseases.

In an exploratory observation that warrants cautious interpretation given the small number of cervical cases, cervical endometriosis appeared to occur more frequently among women aged ≥ 50 years; however, this finding should be considered preliminary and potentially unstable until confirmed in larger studies. Cervical endometriosis in postmenopausal women may represent long-standing disease, effects of hormonal replacement therapy, or residual endometrial tissue implantation from previous instrumentation. This age-related anatomical preference warrants further study to understand the underlying pathological mechanisms and develop appropriate surveillance strategies.

### Endometriotic cysts: Frequency and malignant potential

Endometriotic cysts were observed in 28.8% of our group, with a higher proportion of ovaries involved (*p* < 0.001). This is an important clinical aspect because it brings considerable suffering and increased risk for cancer. Previous studies conducted in South Africa reported similar rates of endometriotic cyst formation (29.4%)^2^; hence, consistency in the frequency of endometriotic cysts across populations can be noted.

The possibility of malignant transformation in endometriotic cysts is a major public health issue. Recent data show a 0.5% – 1% risk of malignant transformation associated with endometriosis; most cancers related to endometriosis are ovarian clear cell and endometrioid adenocarcinomas.^1^ Atypical endometriosis serves as an intermediate precursor lesion, meaning it arises from benign progression through atypical features towards frank malignancy.^13,19^

The local microenvironment in endometriotic cysts, characterised by recurrent bleeding, hemosiderin deposition, iron-catalysed oxidative stress and chronic inflammation, fosters conditions highly conducive to neoplastic transformation.^14^ Understanding this pathophysiology underscores the critical importance of long-term monitoring in women with ovarian endometriomas, particularly when they present with worrisome features such as rapid cyst growth, solid components or atypical imaging characteristics.

### Associated benign, premalignant and malignant lesions

The association of endometriosis with other gynaecological diseases in 38 cases (24.4%) highlights important clinical associations and possible common pathogenetic mechanisms. Uterine leiomyomas were the most common associated lesion (*n* = 18, 47.4%). This reflects the high baseline prevalence of fibroids among women of reproductive age and possible shared aetiological factors such as oestrogen dependence and genetic predisposition.

The presence of adenomyosis in six cases (15.8%) is particularly interesting, as new evidence suggests that adenomyosis and endometriosis may be two forms of the same disease rather than distinct conditions. Both involve ectopic endometrial tissue, have a shared hormonal response system, and often occur together in patients with these conditions.^2^

The association of malignancies and endometriosis may have significant clinical implications. Endometriosis-associated ovarian cancers predominantly comprise the histological subtypes endometrioid and clear cell, which together account for about 76% of all malignancies arising in the context of endometriosis.^1^

Although there were synchronous cases of high-grade intraepithelial lesions and cervical squamous cell carcinoma with endometriosis, no histological or molecular evidence suggested malignant transformation of the endometriotic lesions. Given that cervical squamous cell carcinoma arises through a distinct Human Papillomavirus (HPV)-driven pathway, the coexistence of these lesions is likely coincidental rather than pathogenetically related. In contrast, malignant transformation of endometriosis is most commonly associated with endometrioid and clear cell carcinomas, particularly in ovarian endometriosis. However, their presence underscores the importance of thorough pathological evaluation and the possibility of multiple synchronous gynaecological pathologies.^9^

The association of mature cystic teratomas with endometriosis in two cases is a relatively uncommon but documented phenomenon. These likely represent coincidental findings rather than causally related entities; however, their co-occurrence illustrates the diagnostic complexity of adnexal masses, where multiple pathologies may coexist.^19^

### Diagnostic challenges and clinical practice implications

Endometriosis can be accurately diagnosed histopathologically, provided tissue sampling is adequate, fixation is high quality, and the pathologist has sufficient experience. Patterns showing only one or two diagnostic components, especially stromal endometriosis without any glandular elements, present considerable interpretive problems and may be easily misdiagnosed as benign or malignant conditions, such as Kaposi sarcoma, low-grade ESS or adenocarcinoma.^2^

Immunohistochemical characterisation is an important part of the diagnostic workup of tumours with endometrial stromal differentiation, particularly when morphology alone is not sufficient to make a definitive diagnosis. CD10 is a cell membrane neutral endopeptidase and is a relatively sensitive marker of endometrial stromal differentiation. It is routinely used to confirm the stromal lineage. Expression of CD10 is not entirely specific, however, as it is known to be expressed in a number of other neoplasms. In the appropriate morphological and clinical setting, positivity strongly suggests stromal differentiation. Besides CD10 immunoreactivity, the presence of oestrogen receptor (ER) and progesterone receptor (PR) expression confirms hormonal responsiveness of the lesional cells, which is a hallmark of endometrial stroma and not only supports the diagnosis but also has direct therapeutic implications considering the role of hormone deprivation therapy in the management of endometrial stromal sarcoma (ESS). This immunohistochemical panel, as a group, represents best practice in diagnostically difficult cases, providing an objective adjunct to haematoxylin and eosin morphology.^20,21^ Adequate clinical information about the patient’s age and symptoms, as well as surgical findings and imaging characteristics, will also increase diagnostic accuracy by informing pathological interpretation and raising suspicion for endometriosis in atypical presentations.^22^ Because clinical presentation and imaging supported the diagnosis of stromal endometriosis, immunohistochemistry was not required in cases in our study.

This study’s findings have several important clinical practice implications in resource-limited settings. Firstly, the relatively low diagnostic frequency may indicate underdiagnosis of endometriosis in our population; hence, enhanced clinician awareness is needed, along with improved access to laparoscopic evaluation and investment in specialised gynaecological pathology services. Secondly, since a substantial proportion of extragonadal endometriosis occurs particularly in surgical scars and the umbilicus, clinical vigilance is required with a heightened index of suspicion for this condition among women who present with cyclic pain or masses at these sites.

Thirdly, the finding of endometriosis in postmenopausal women, particularly with cervical involvement, reinforces that endometriosis should stay in the differential diagnosis throughout the entire female lifespan. Fourthly, the coexistence of premalignant and malignant lesions underscores the importance of thorough pathological evaluation, long-term monitoring of endometriomas and multidisciplinary management for complicated cases. Finally, the diverse histopathological patterns observed in this cohort highlight the potential for diagnostic difficulty, particularly in atypical or extra-pelvic lesions. These findings support continued training in the histopathological diagnosis of endometriosis and the selective use of ancillary techniques and specialist consultation in diagnostically challenging cases.

### Limitations and future directions

The limitations inherent in single-institution retrospective studies include selection bias, incomplete clinical data for some cases and limited generalizability to other populations. Critically, because the denominator comprises only specimens submitted for histopathological examination, this study is subject to pathology-denominator bias: Women without surgical intervention or histopathological work-up are excluded, so the calculated diagnostic frequency cannot be extrapolated to estimate true population prevalence. The relatively small sample size may have limited statistical power to detect associations between endometriosis patterns and demographic or clinical variables. Our study could not capture the complete range of endometriosis severity or relate histopathological findings to laparoscopic stage, symptom intensity or treatment results. Future prospective, multi-centre studies with larger sample sizes, clinically well-characterised and molecularly profiled would provide more substantial evidence on the epidemiology, pathogenesis and clinical course of endometriosis in African populations.

## Conclusion

In this retrospective 10-year study, we analysed 156 cases of histopathologically confirmed endometriosis, revealing a low diagnosis frequency that likely reflects underdiagnosis rather than rarity. The ovary was the most common site (36.5%), followed by the umbilicus (22.4%). A significant link was noted between cervical endometriosis and patients aged ≥ 50 years, although based on a small number of cases (*p* = 0.008). Endometriotic cysts were found in 28.8% of cases, predominantly ovarian (*p* = 0.001). Associated lesions were identified in 38/156 cases (24.4%), including benign, premalignant and malignant lesions. Malignant lesions were present in 4/156 cases (2.6%), highlighting the necessity for thorough histopathological evaluation and long-term monitoring.

This study’s findings suggest that endometriosis may be substantially underdiagnosed in African contexts, and support clinicians maintaining a high index of suspicion for the disease across all age groups, including post-menopausal women; larger, prospective, population-based studies are needed to confirm the extent of underdiagnosis. Histopathological confirmation remains the diagnostic gold standard, so carefully evaluate all resected specimens because premalignant and malignant pathologies may coexist in a notable percentage of cases.
